# The potential causal relationship between various lifestyles and depression: a univariable and multivariable Mendelian randomization study

**DOI:** 10.3389/fpsyt.2024.1343132

**Published:** 2024-02-29

**Authors:** Shaobo Guo, Wenhui Zhu, Likai Yu, Lishi Jie, Di Tian, Tianci Zhao, Biqing Zhao, Biao Zhang

**Affiliations:** ^1^ The Affiliated Hospital of Nanjing University of Chinese Medicine, Department of Geriatrics, Nanjing, China; ^2^ Key Laboratory for Metabolic Diseases in Chinese Medicine, First College of Clinical Medicine, Nanjing University of Chinese Medicine, Nanjing, China; ^3^ Jiangsu Province Hospital of Chinese Medicine, Department of Geriatrics, Nanjing, China; ^4^ The Affiliated Hospital of Nanjing University of Chinese Medicine, Department of Orthopedics, Nanjing, China

**Keywords:** depression, lifestyle, Mendelian randomization, genome-wide association study, causality

## Abstract

**Background:**

Previous studies have shown that lifestyle was associated with depression. Thus, the aim of this study was to examine the causality between multiple lifestyles and depression by Mendelian randomization (MR) analysis.

**Methods:**

The single-nucleotide polymorphisms (SNPs) of depression, alcoholic drinks per week, sleeplessness or insomnia, body mass index (BMI), mood swings, weekly usage of mobile phone in the last 3 months, beef intake, cooked vegetable intake, and “smoking status: never” were acquired from the Integrative Epidemiology Unit Open genome-wide association study database. Causal effects of eight exposure factors and depression were investigated using MR-Egger, weighted median, inverse variance weighted (IVW), simple mode, and weighted mode, and results were primarily referred to IVW. Subsequently, univariable MR (UVMR) analysis was performed on eight exposure factors and depression, separately. In addition, sensitivity analysis, including heterogeneity test, horizontal pleiotropy, and leave-one-out (LOO) methods, was conducted to evaluate the stability of MR results. Furthermore, multivariable MR (MVMR) analysis was carried out.

**Results:**

UVMR analysis revealed that all eight exposure factors were causally associated with depression; alcoholic drinks per week, sleeplessness or insomnia, BMI, mood swings, weekly usage of mobile phone in the last 3 months, and cooked vegetable intake were risk factors, and beef intake and “smoking status: never” were protection factors. Heterogeneity tests revealed no heterogeneity for alcoholic drinks per week, sleeplessness or insomnia, mood swings, weekly usage of mobile phone in the last 3 months, and cooked vegetable intake. Meanwhile, there was no horizontal pleiotropy in UVMR, and LOO analysis verified that univariable analysis results were reliable. Moreover, MVMR analysis indicated that mood swings and weekly usage of mobile phone in the last 3 months were risk factors, and beef intake was a protection factor for depression when multiple factors occurred at the same time.

**Conclusion:**

Alcoholic drinks per week, sleeplessness or insomnia, BMI, mood swings, weekly usage of mobile phone in the last 3 months, and cooked vegetable intake were risk factors, and beef intake and “smoking status: never” were protection factors. In addition, mood swings, weekly usage of mobile phone in the last 3 months, and beef intake had a direct effect on depression when multiple factors occurred simultaneously.

## 1 Introduction

Depression is a common illness that significantly limits social and psychological functioning and reduces the quality of life (1). More than 300 million people worldwide suffer from depression, making it a significant public health issue that imposes a global burden (2, 3). Depression was ranked as the fourth-leading cause of disease burden in 2000 (4). Mild depression manifests as persistent sadness, loss of interest or pleasure, and feelings of worthlessness. Severe depression, on the other hand, is characterized by recurrent suicidal tendencies (5).

The interplay of genes, biological factors, and the environment leads to the onset of depression (6). Compared to men, women have a higher prevalence of depression (7). Lifestyle is closely related to health; a healthy lifestyle promotes feelings of happiness and reduces feelings of displeasure and misery. Analyses of the correlation between lifestyle and the incidence of depressive symptoms suggest that lifestyle and its various dimensions are related to the onset of depressive symptoms (8). Excessive alcohol consumption increases the risk of depression (9). However, moderate drinking can mitigate or suppress the impact of stress on depression (10). Smoking increases the risk of developing depression (11). Diet and health are closely related, and healthy eating habits can reduce the risk of depression (12). Additionally, there is a close relationship between body mass index (BMI) and depression, with obese individuals being more prone to depression (13). Sleep disturbances are linked to many mental illnesses. Insomnia is a risk factor for depression (14), and sleep interventions can be beneficial in alleviating clinical depressive symptoms (15). Studies have found that frequent mobile phone use is a risk factor for adverse mental health outcomes in young people over a 1-year follow-up (16).

Mendelian randomization (MR) is a method that uses genetic instrumental variables (IVs) to assess the causal direction between exposure and outcome, and it is unaffected by confounding factors and reverse causality (17, 18). In the face of clinical research where randomized controlled trials (RCTs) are challenging to conduct, including but not limited to impractical and unethical RCT studies, MR can strengthen the inference of direct causal relationships between exposure factors and diseases, while avoiding difficult RCT studies (17). Recently, there has been a surge in MR articles related to depression, but most of the content focuses on the application of biomarkers, with few causal studies on various lifestyle factors and depression. In particular, there is a lack of multivariate MR (MVMR) research on the role of various lifestyle factors in depression (19). Therefore, this paper is devoted to study the causal relationships of various lifestyles and depression by means of univariate and multivariate methods. The univariable MR (UVMR) analysis evaluates the influence of a single predictor variable on the outcome (20). MVMR is an extension of UVMR that estimates the direct causal effect of each exposure factor on the outcome, taking into account the pleiotropy among multiple variables (21).

This study, based on single-nucleotide polymorphism (SNP) data of depression and exposure factors (eight lifestyle factors) from public databases, employed both UVMR and MVMR methods to investigate the causal relationship between different lifestyles and depression. Sensitivity analyses were conducted to assess the impact of assumptions on the study findings and to ensure the robustness of the results. From a genetic perspective, this research explored the potential roles of various lifestyles in the progression of depression.

## 2 Materials and methods

### 2.1 Source of data

The datasets of depression (finn-b-F5_ DEPRESSIO), alcoholic drinks per week (ieu-b-73), sleeplessness or insomnia (ukb-a-13), BMI (ukb-a-248), mood swings (ebi-a-GCST006944), weekly usage of mobile phone in the last 3 months (ukb-b-17999), beef intake (ukb-b-2862), cooked vegetable intake (ukb-b-8089), and “smoking status: never” (ukb-d-20116_0) were retrieved from the Integrative Epidemiology Unit (IEU) Open Genome-Wide Association Study (GWAS) database (https://gwas.mrcieu.ac.uk/). The finn-b-F5_ DEPRESSIO dataset included 16,380,457 SNPs from 215,644 samples. The ieu-b-73 dataset included 11,887,865 SNPs from 335,394 samples. The ukb-a-13 dataset included 10,894,596 SNPs from 336,965 samples. The ukb-a-248 dataset included 10,894,596 SNPs from 336,107 samples. The ebi-a-GCST006944 dataset included 10,894,596 SNPs from 329,428 samples. The ukb-b-17999 dataset included 9,851,867 SNPs from 386,626 samples. The ukb-b-2862 dataset included 9,851,867 SNPs from 461,053 samples. The ukb-b-8089 dataset included 9,851,867 SNPs from 448,651 samples. The ukb-d-20116_0 dataset included 13,586,591 SNPs from 359,706 samples. Details of the above datasets are given in **Table 1**.

**Table 1:** Detailed information about the datasets.

Trait	Year	Population	GWAS ID	Sample size	SNP	Consortium
Depression	2021	European	finn-b-F5_DEPRESSIO	215,644	16,380,457	FinnGen
Alcoholic drinks per week	2019	European	ieu-b-73	335,394	11,887,865	GWAS and Sequencing Consortium of Alcohol and Nicotine use
Sleeplessness/insomnia	2017	European	ukb-a-13	336,965	10,894,596	UK Biobank
Body mass index (BMI)	2017	European	ukb-a-248	336,107	10,894,596	UK Biobank
Mood swings	2017	European	ebi-a-GCST006944	329,428	10,894,596	EBI
Weekly usage of mobile phone in the last 3 months	2018	European	ukb-b-17999	386,626	9,851,867	UK Biobank
Beef intake	2018	European	ukb-b-2862	461,053	9,851,867	UK Biobank
Cooked vegetable intake	2018	European	ukb-b-8089	448,651	9,851,867	UK Biobank
Smoking status: Never	2018	European	ukb-d-20116_0	359,706	13,586,591	UK Biobank

### 2.2 Pre-processing of data

The adjusted *p-*values of the forward and reverse MR analysis results were calculated by the false discovery rate (FDR) method. The reading and filtering of exposure factors was carried out via the extract_instruments function of the TwoSampleMR package (*p* < 5×10^−8^) (22) in forward MR analysis. In the reverse MR analysis, IVs with significant correlation with exposure factors were found through *p* < 5×10^−6^. IVs were removed using linkage disequilibrium analysis (LDA) (*r* ^2 = ^0.001 and kb = 10,000). Three basic premises underlie UVMR studies (1): a robust and significant correlation existed between exposures and IVs (2); IVs were not related to confounding factors; and (3) IVs could only influence outcomes through exposure and not through other channels. Three hypotheses of the MVMR require consideration of all exposures: (1) in the case of other exposures given, SNPs were strongly correlated with the remaining exposures. (2) In conditions that give all exposures, SNPs and outcomes were independent. (3) SNPs were independent of all confounding (23).

### 2.3 UVMR analysis and sensitivity analysis

Firstly, genetic correlation between exposure factors and outcome was calculated using the ldscr package. Then, five diverse MR methods, MR-Egger (24), weighted median (25), inverse variance weighted (IVW) (26), simple mode, and weighted mode (22, 27), were adopted to explore the causality of eight exposure factors with depression, the most important of which was IVW. In addition, odds ratio (OR) was calculated, and OR > 1 indicated that exposure factor was a risk factor, while OR < 1 revealed that exposure factor was a protection factor. Results were presented using scatter plots, forest plots, and funnel plots. Moreover, sensitivity analysis was conducted to evaluate the reliability of UVMR results. First, heterogeneity test was conducted by Cochran’s *Q* test. Namely, there was no heterogeneity when the *p-*value was greater than 0.05 in Cochran’s *Q* test. If there was heterogeneity (*p* < 0.05), the IVW test was performed for random effects. Secondly, the horizontal pleiotropy test was performed via TwoSampleMR function mr_pleiotropy_test in *R*, and if *p* > 0.05, it indicated no horizontal pleiotropy, meaning that there were no confounding factors in the study. In addition, horizontal pleiotropy was further validated using MR-Egger analysis (28). Finally, leave-one-out (LOO) analysis was conducted by gradually eliminating each SNP, and if the effect of the remaining SNPs on the outcome variable did not change significantly, this indicates that the results of MR analysis was reliable.

### 2.4 Multivariable MR analysis

In order to detect causal relations between eight exposure factors and depression at the multivariate level, the TwoSampleMR package was utilized to harmonize effect equivalents and effect sizes followed by MVMR analysis. Multiple exposure factor IVs were filtered by the mv_lasso_feature_selection function. Then, ORs were computed as before.

## 3 Results

### 3.1 Genetic correlation

The gene correlation between eight exposure factors and outcome was calculated. The results revealed that BMI (*r*_g_ = 0.102, *p* < 0.001), sleeplessness_insomnia (*r*_g_ = 0.372, *p* < 0.001), mood swings (*r*_g_ = 0.318, *p* = 0.002), weekly usage of mobile phone in the last 3 months (*r*_g_ = 0.209, *p* < 0.001), alcoholic drinks per week (*r*_g_ = 0.118, *p* < 0.001), and cooked vegetable intake (*r*_g_ = 0.0579, *p* = 0.049) and depression had positive genetic correlations, while beef intake (*r*_g_ = −0.171, *p* < 0.001) and “smoking status: never” (*r*_g_ = −0.324, *p*< 0.001) showed an inverse genetic association.

### 3.2 Eight exposure factors were significantly causally associated with depression

**Figure 1:**
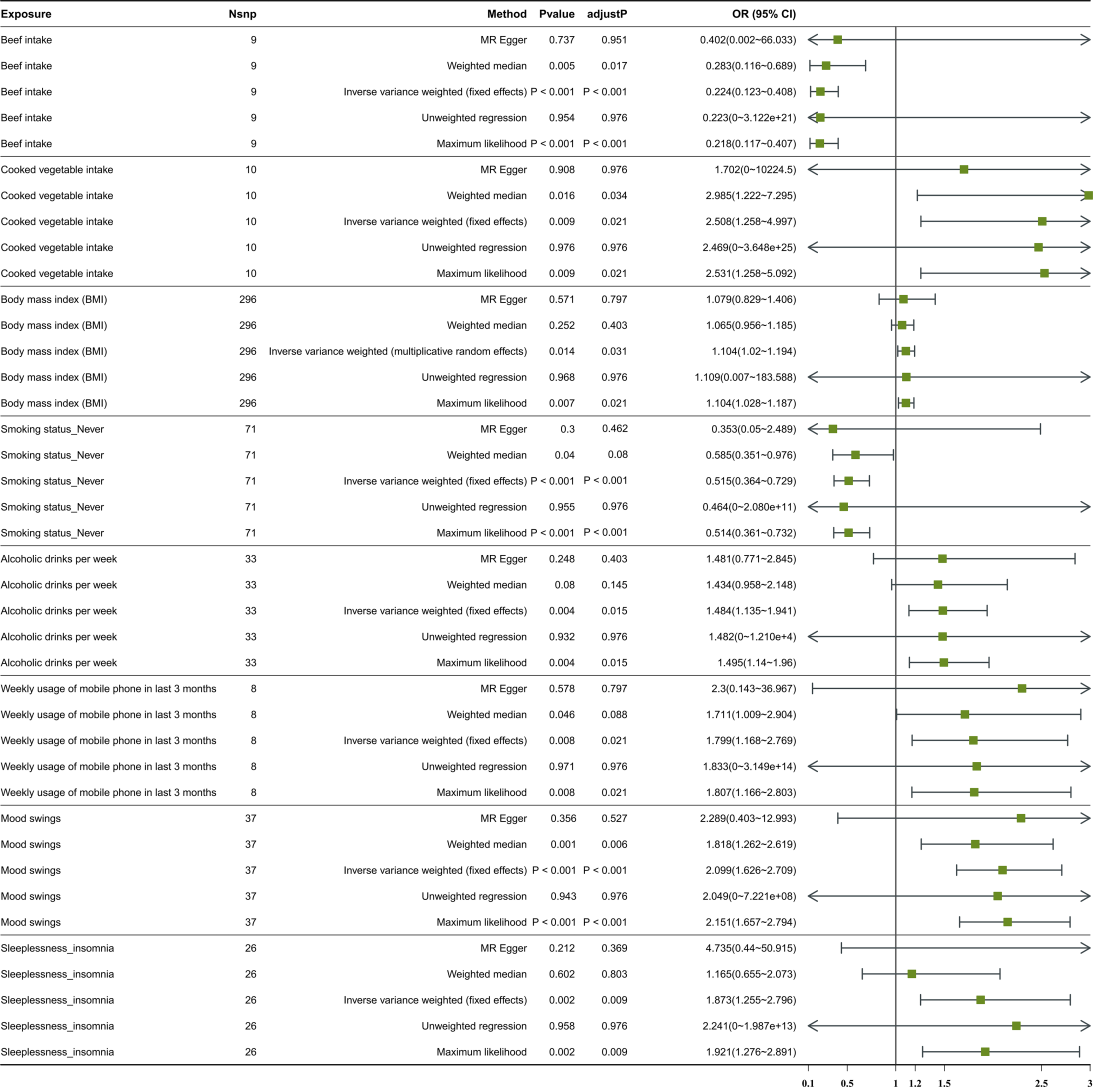
The forest plot of studies that evaluated the causal effect between exposures and depression using values obtained by the IVW MR method. Nsnp stands for the number of SNPs. An OR value greater than 1 indicates a risk factor, whereas a value less than 1 suggests a protective factor. This is in reference to a binary variable. IVW, inverse variance weighted; MR, Mendelian randomization; SNPs, single-nucleotide polymorphisms; OR, odds ratio; CI, confidence interval.

**Figure 2:**
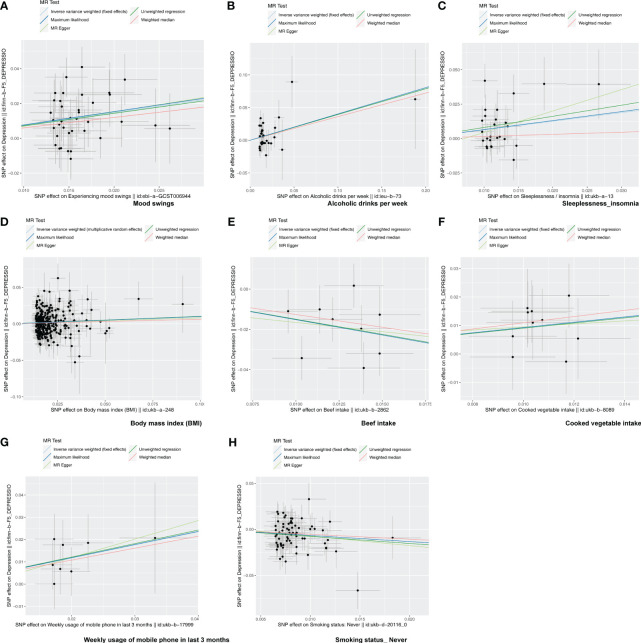
The scatter plot of MR analysis. **(A–H)** Scatter plots for the causal association between depression and mood swings **(A)**, alcoholic drinks per week **(B)**, sleeplessness/insomnia **(C)**, body mass index **(D)**, beef intake **(E)**, cooked vegetable intake **(F)**, weekly usage of mobile phone in the last 3 months **(G)**, and “smoking status: never” **(H)**. Each point on the graph represents an SNP locus. The *x*-axis represents the effect of the SNP on the exposure, while the *y*-axis represents the effect of the SNP on the outcome. The colored lines represent the fit results of different MR algorithms. A positive slope of the line indicates a risk factor, while a negative slope indicates a protective factor.

**Figure 3:**
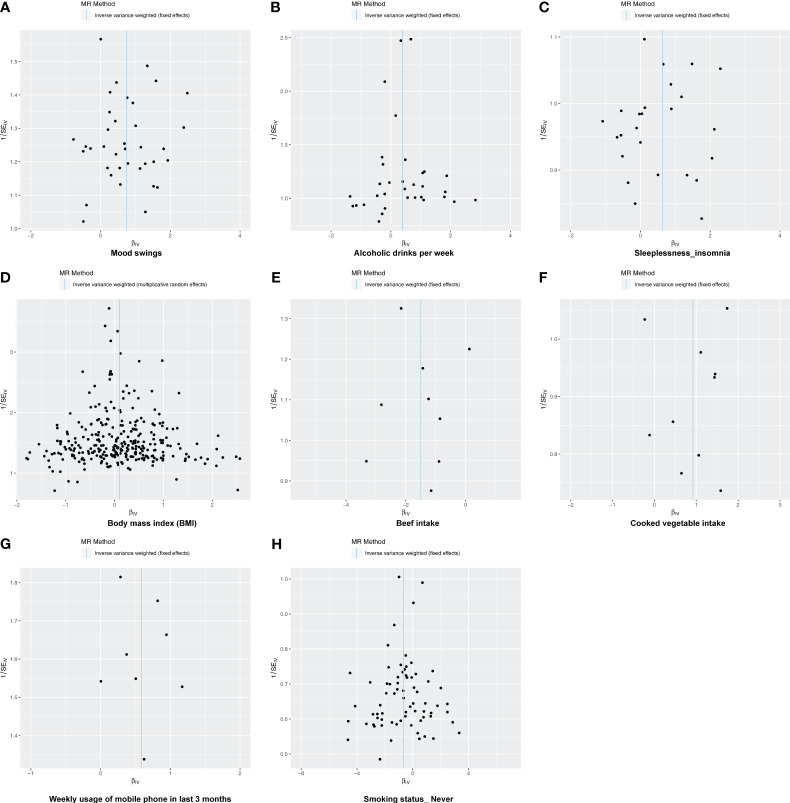
The funnel plot of MR analysis. **(A–H)** Funnel plots to show the overall heterogeneity of MR analysis for the effect of mood swings **(A)**, alcoholic drinks per week **(B)**, sleeplessness/insomnia **(C)**, body mass index **(D)**, beef intake **(E)**, cooked vegetable intake **(F)**, weekly usage of mobile phone in the last 3 months **(G)**, and “smoking status: never” **(H)** on depression. If samples are symmetrically distributed on both sides of the IVW line, then the MR conforms to Mendel’s second law of random assortment.

**Figure 4:**
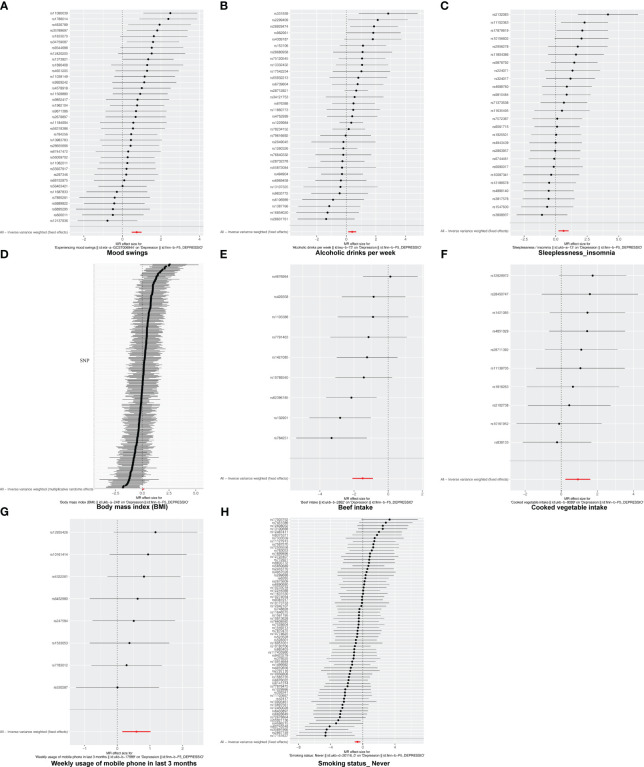
The forest plot of studies that determined the diagnostic efficacy between exposures and depression using values obtained by the IVW MR method. **(A)** Mood swings; **(B)** alcoholic drinks per week; **(C)** sleeplessness/insomnia; **(D)** body mass index; **(E)** beef intake; **(F)** cooked vegetable intake; **(G)** weekly usage of mobile phone in the last 3 months; **(H)** “smoking status: never”. Solid points on the left indicate a reduction, while those on the right indicate an increase.

Following screening, 34 IVs for alcoholic drinks per week, 28 IVs for sleeplessness or insomnia, 297 IVs for BMI, 40 IVs for mood swings, 10 IVs for weekly usage of mobile phone in the last 3 months, 14 IVs for beef intake, 11 IVs for cooked vegetable intake, and 76 IVs for “smoking status: never” were obtained (**Supplementary Tables 1**–**8**). The IVW method revealed a causal relationship between alcoholic drinks per week (adj.*p* = 0.015, OR = 1.484), sleeplessness or insomnia (adj.*p* = 0.009, OR = 1.873), BMI (adj.*p* = 0.031, OR = 1.104), mood swings (adj.*p* < 0.001, OR = 2.099), weekly usage of mobile phone in the last 3 months (adj.*p* = 0.021, OR = 1.799), cooked vegetable intake (adj.*p* = 0.021, OR = 2.508), beef intake (adj.*p* < 0.001, OR = 0.224), and “smoking status: never” (adj.*p* < 0.001, OR = 0.515) and depression (**Figure 1**). Scatter plots for alcoholic drinks per week, sleeplessness or insomnia, BMI, mood swings, weekly usage of mobile phone in the last 3 months, and cooked vegetable intake had positive slopes, suggesting that they were risk factors, while beef intake and “smoking status: never” had negative slopes, indicating that they were protection factors for depression (**Figures 2A–H**). The symmetrical distribution of the samples along both sides of the IVW line in the funnel plot revealed that UVMR analysis results conforms to the second law of MR grouping (**Figures 3A–H**). The results of forest plots were consistent with the previous results (**Figures 4A–H**).

### 3.3 Sensitivity analysis of eight exposure factors revealed UVMR analysis results were reliable

**Figure 5:**
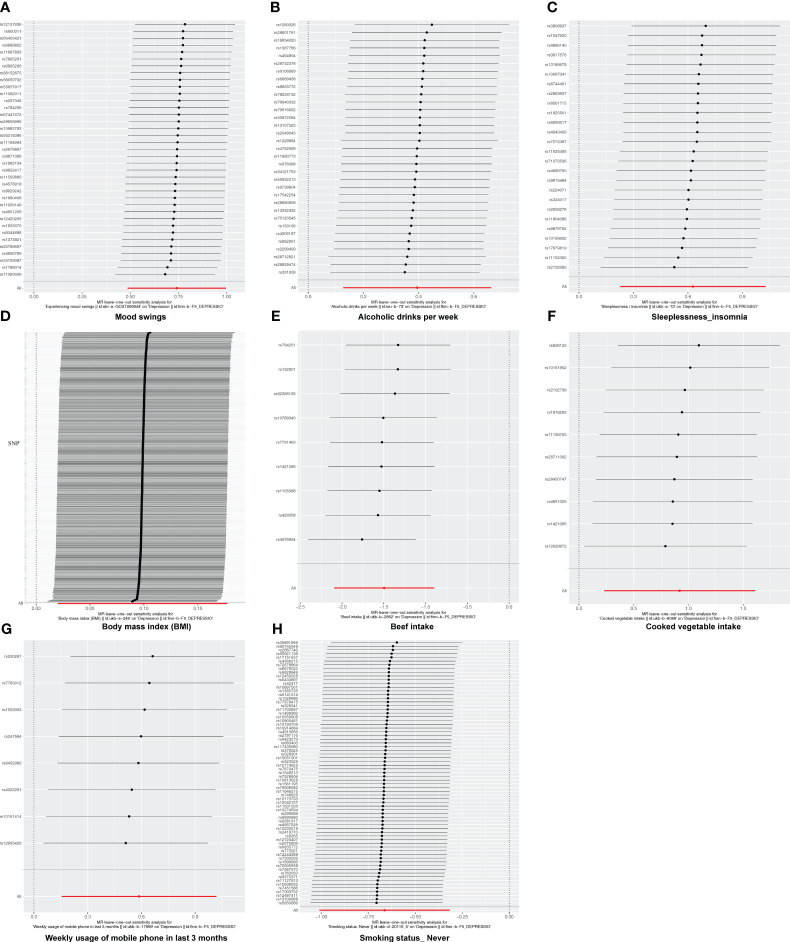
The leave-one-out analysis of MR analysis. **(A)** Mood swings; **(B)** alcoholic drinks per week; **(C)** sleeplessness/insomnia; **(D)** body mass index; **(E)** beef intake; **(F)** cooked vegetable intake; **(G)** weekly usage of mobile phone in the last 3 months; **(H)** “smoking status: never”.

In this study, there was no heterogeneity for alcoholic drinks per week (*p* = 0.070), sleeplessness or insomnia (*p* = 0.106), mood swings (*p* = 0.129), weekly usage of mobile phone in the last 3 months (*p* = 0.267), and cooked vegetable intake (*p* = 0.902) as exposure factors, respectively (**Table 2**). The heterogeneity test results for BMI (*p* = 0.006), beef intake (*p* = 0.022), and “smoking status: never” (*p* = 0.002) showed that *p* was less than 0.05 in Cochran’s *Q* test, but *p* < 0.05 in IVW and so it did not affect the results (**Table 1**) and all *p*-values in MR-Egger analysis were greater than 0.05 (**Figure 1**). Horizontal pleiotropy showed that there was no horizontal pleiotropy between eight exposure factors and depression (*p* > 0.05, **Table 3**). Furthermore, there were no points of serious bias in the results of LOO analysis, indicating that the results were reliable (**Figures 5A–H**). Thus, eight exposure factors, alcoholic drinks per week, sleeplessness or insomnia, BMI, mood swings, weekly usage of mobile phone in the last 3 months, beef intake, cooked vegetable intake, and “smoking status: never”, were causally associated with the occurrence of depression.

**Table 2:** Heterogeneity test between eight exposure factors and depression.

Outcome	Exposure	*Q*	*Q*_df	*Q*_pval
Depression	Beef intake	25.2289	13.0000	0.0215
Depression	Cooked vegetable intake	4.8372	10.0000	0.9018
Depression	Body mass index (BMI)	360.9072	296.0000	0.0058
Depression	Smoking status: Never	115.5257	75.0000	0.0018
Depression	Alcoholic drinks per week	45.7001	33.0000	0.0697
Depression	Weekly usage of mobile phone in the last 3 months	11.1234	9.0000	0.2673
Depression	Mood swings	49.0984	39.0000	0.1289
Depression	Sleeplessness_insomnia	36.4613	27.0000	0.1056

**Table 3:** Horizontal pleiotropy test between eight exposure factors and depression.

Outcome	Exposure	Egger_intercept	SE	*Q*_pval
Depression	Beef intake	0.0137	0.0293	0.6499
Depression	Cooked vegetable intake	−0.0140	0.0432	0.7537
Depression	Body mass index (BMI)	−0.0003	0.0024	0.8885
Depression	Smoking status: Never	0.0073	0.0076	0.3370
Depression	Alcoholic drinks per week	−0.0009	0.0063	0.8854
Depression	Weekly usage of mobile phone in the last 3 months	−0.0007	0.0280	0.9802
Depression	Mood swings	−0.0103	0.0144	0.4755
Depression	Sleeplessness_insomnia	0.0017	0.0078	0.8335

### 3.4 No causal relationship between depression and eight exposure factors based on reverse MR analysis

Since the relationship between exposure factors and outcome was not one-way and may be two-way, reverse MR analysis was performed to further confirm the causal relationship between depression and eight lifestyle types. The results revealed that there was no significant causal relationship between the eight outcomes and exposure factor (depression), which further confirmed the relationship between the eight lifestyles and the risk of depression (**Supplementary Figure 1**).

### 3.5 Mood swings, weekly usage of mobile phone in the last 3 months, and beef intake had a direct effect on depression when multiple factors occurred simultaneously

**Figure 6:**
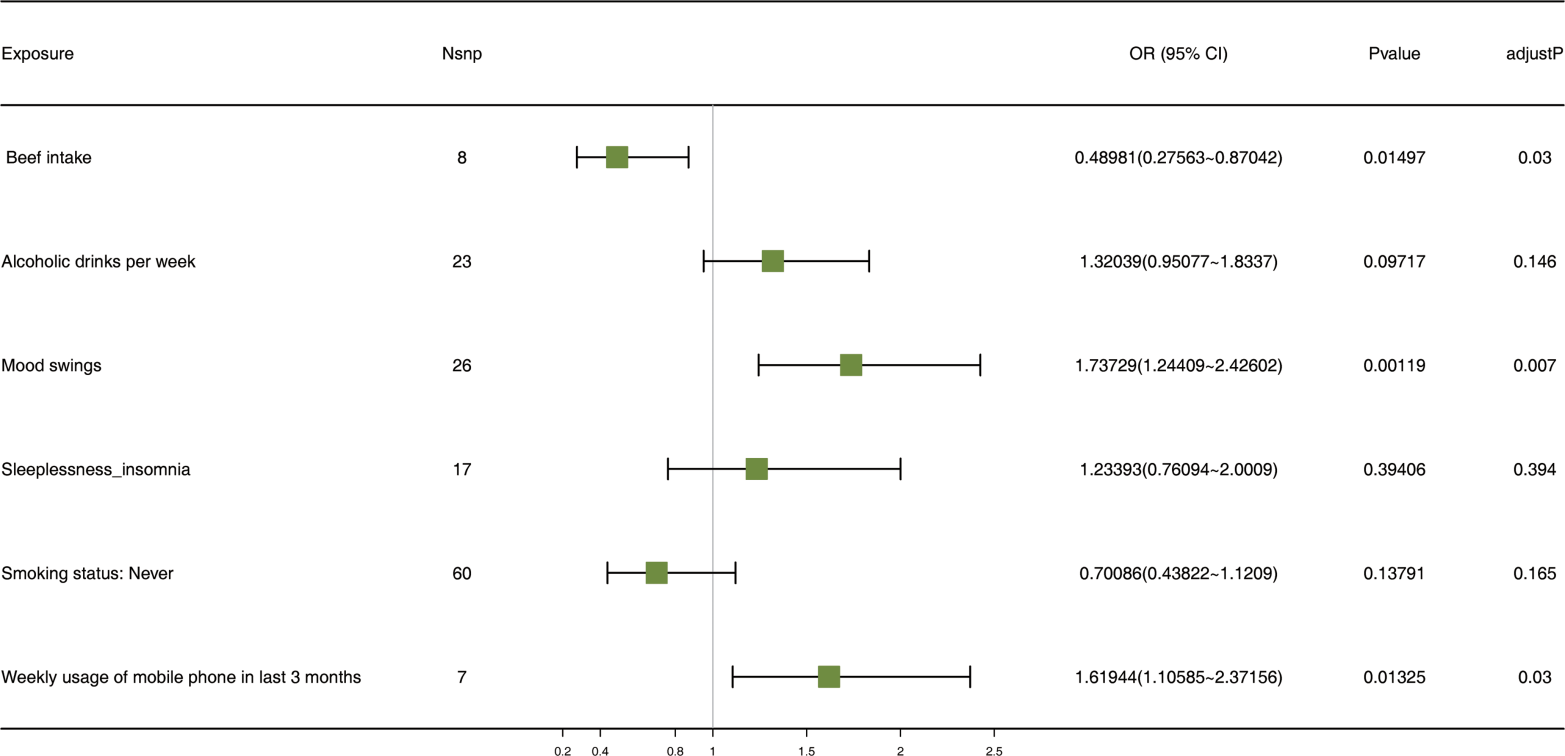
The forest plot of MVMR analysis. Nsnp stands for the number of SNPs. An OR value greater than 1 indicates a risk factor, whereas a value less than 1 suggests a protective factor. This is in reference to a binary variable. MVMR: multivariate Mendelian randomization; SNPs: single-nucleotide polymorphisms; OR, odds ratio; CI: confidence interval.

Following screening, 141 SNPs from six exposure factors, alcoholic drinks per week, sleeplessness or insomnia, mood swings, weekly usage of mobile phone in the last 3 months, beef intake, and “smoking status: never”, were utilized as IVs for the MVMR analysis. MVMR analysis suggested that mood swings (*p* = 0.001, OR = 1.737) and weekly usage of mobile phone in the last 3 months (*p* = 0.013, OR = 1.619) were risk factors and beef intake (*p* = 0.015, OR = 0.490) was a protection factor. Moreover, mood swings, weekly usage of mobile phone in the last 3 months and beef intake had a direct effect on depression when multiple factors occurred simultaneously (**Figure 6**).

## 4 Discussion

Depression is a global disease, greatly exacerbating socio-economic burdens (1, 2, 29). Healthy lifestyle habits help reduce the risk of depression. MR treats genetic variations as natural experiments to evaluate the causal relationship between two variables (30). We assessed the causal relationship between eight different lifestyles and depression through UVMR and MVMR.

In this study, we observed a causal relationship between multiple lifestyle-related exposure factors and depression. The UVMR analysis showed that alcoholic drinks per week, sleeplessness or insomnia, BMI, mood swings, weekly usage of mobile phones in the last 3 months, and cooked vegetable intake were risk factors, while beef intake and never smoking were protective factors.

Prospective studies have shown deep connections between alcohol consumption and smoking with depression (31, 32). One study showed that alcohol consumption and headache were negatively correlated and that the comorbidity of headache with depression was common (33). A study of 6,646 ordinary people over 3 and 6 years found that reducing smoking and alcohol consumption were associated with improvements in depressive symptoms through multiple cross-sectional analyses (34). Risky drinking and alcohol abuse may increase the risk of depression (9). However, surveys indicate that moderate alcohol intake (5–15 g/day) can reduce the risk of developing depression (35, 36). Some drugs that treat depression can also treat alcohol dependence (37). SEMA3A may be a common genetic risk gene for alcohol dependence and depression (38). There is a potential bidirectional relationship between smoking and depression: smokers may have an increased risk of developing depression, and among those with depression, the smoking rate is higher than among non-smokers. Cross-sectional studies show that smokers are more likely to develop depression, and a higher proportion of people with depression smoke (31). Longitudinal studies suggest that smoking is a potential factor for developing depression (39). Recent MR studies indicate that smoking is a causal risk factor for diagnosed depression (40), and inflammation is likely involved in at least part of the connection between smoking and depression (41). Our UVMR showed a causal relationship between weekly alcohol consumption and never smoking with depression. Combined with MVMR, weekly drinking is a risk factor, while never smoking is a protective factor.

The relationship between diet and depression is profound. Healthy dietary habits and avoiding pro-inflammatory foods can prevent depression to a certain extent (12). Multiple studies suggest that the Mediterranean diet can reduce the risk of depression (12, 42, 43). The Mediterranean diet emphasizes the consumption of plant-based foods rich in fruits, vegetables, whole grains, nuts, seeds, and legumes (44). Interestingly, the intake of raw vegetables seems to have a more significant effect on mental health than cooked vegetables (45). Through a UVMR analysis, we found a direct causal relationship between cooked vegetables and depression. Intriguingly, cooked vegetables act as a risk factor for depression. This might be because cooking can destroy some beneficial components in vegetables, such as polyphenols, which are known to alleviate depression (46, 47). Meat intake is an integral part of the Mediterranean diet (44). Our UVMR and MVMR analyses indicate a direct causal relationship between beef intake and depression, with beef acting as a protective factor. Other MR studies support this viewpoint (48). Interestingly, several studies suggest that excessive beef consumption is linked to an increased risk of depression (49). It is undeniable that beef contains vitamins and minerals that have a positive effect on mood. Thus, moderate beef intake might have beneficial effects on reducing the risk of depression. The Mediterranean diet helps maintain a healthy weight range. An abnormal BMI is closely linked to depression. Individuals with metabolic health issues have a higher risk of depression than those without such issues (50, 51). A joint survey questionnaire of 18,025 participants showed that the prevalence of depression was highest among those with unhealthy metabolic obesity, accompanied by an abnormal elevation of C-reactive protein. This seems to indicate that inflammation caused by metabolic anomalies might increase the risk of depression (52). A longitudinal study also linked the inflammatory consequences of obesity to depression (53). However, through our MR analysis, there is a direct causal relationship between BMI and depression. An MR study showed that being overweight does not increase the risk of depression due to inflammation marked by an abnormal C-reactive protein caused by metabolic abnormalities. Instead, being overweight directly increases the risk of depression, fully supporting our analysis (54).

Lifestyle habits are closely linked to depression. With the increasing prevalence and frequency of smartphone use, the relationship between smartphones and depression has attracted widespread attention and research. Overuse of phones might elevate the risk of developing depression (55). Excessive smartphone use can increase transient negative emotions in users, and repeated or persistent negative emotions can heighten the risk of depression. Reducing smartphone usage might have long-term positive effects (56). Another piece of evidence suggests that frequent phone use can decrease physical activity in users, thereby raising the risk of depression (57, 58). Prolific smartphone use can also deteriorate sleep quality, indirectly increasing the risk of depression (59). A study indicates that over-reliance on smartphones might be a strategy driven by an escapist motive linked to depression (60). However, most studies focus primarily on adolescent populations, and many of the findings are indirect, which imposes certain limitations on the research methods. Our UVMR analysis indicates a direct causal relationship between excessive smartphone use and depression. Sleep-related characteristics are connected to depression. There might be a unidirectional relationship between sleep disturbances and depression, meaning that insomnia might precede depression (61). A meta-analysis suggests that sleep disturbances could be a harbinger of depression and may serve as one of its risk factors (62). The process through which insomnia affects depression might begin with alterations in the arousal system, affecting biological substrates related to emotional processing, eventually influencing cognitive systems and leading to depression (63, 64). Another piece of evidence posits that treating insomnia symptoms in depression patients can alleviate their depressive symptoms (65). A separate MR study reveals that insomnia might have a causal relationship with coronary artery disease, depressive symptoms, and subjective wellbeing (66). Diurnal mood variations, characterized by worse moods upon waking and improved moods in the evening, are a core feature of depression (67, 68). Neuroendocrine measurements have shown that depressed patients exhibit significantly elevated levels of cortisol and adrenocorticotropic hormone in the morning (69), potentially linked to the diurnal mood fluctuations seen in depression. Another finding attributes these mood fluctuations in depression to diurnal metabolic activities in areas like the ventrolateral–prefrontal, parietal, temporal, and frontal regions, as well as the cerebellum (70). Our results obtained from the UVMR analysis have further confirmed that mood swings are a significant risk factor for depression. This discovery enhances our understanding of the relationship between mood swings and depression, providing a valuable scientific basis for more in-depth research into this complex relationship.

Further MVMR analysis indicates that after considering behaviors such as alcoholic drinks per week, sleeplessness or insomnia, mood swings, weekly usage of mobile phone in the last 3 months, beef intake, and never smoking status, mood swings, weekly usage of mobile phone in the last 3 months, and beef intake have a strong causal relationship with depression. Our research findings are consistent with previous studies, which showcased the relationship between mood swings, weekly mobile phone usage in the last 3 months, and beef intake with depression (48, 55, 71). Healthy lifestyle habits can help reduce the risk of depression. Increased phone usage can lead to reduced sleep duration; hence, limiting phone usage among adolescents can help reduce insomnia, subsequently lowering the risk of depression (72). Mood swings are related to sleep duration (67), with the correlation of IL6 being significantly associated with emotional ratings (73). For those with depression, sleep deprivation is more likely to intensify diurnal mood fluctuations (74), which could further elevate the risk of depression. Healthy dietary habits help in reducing the risk of depression. While beef intake is essential for those with depression, it is crucial to maintain it within a certain limit. Overconsumption of beef might actually exacerbate the risk of depression (49).

## 5 Conclusion

Our study indicates that various lifestyle factors have both univariable and multivariable causal relationships with depression, and we have drawn corresponding conclusions. Our analysis focusing on dietary habits primarily targets the European population, and comparatively, there is a lack of analysis concerning the dietary habits of the Asian population. Furthermore, in exploring the causal relationship between cooked vegetable intake and beef intake with depression, we cannot completely rule out the influence of cultural differences on the results. In future studies, we will consider more deeply the impact of cultural factors on depression. The sample population for mobile phone usage frequency is mostly adolescent depression patients, and there is a relative scarcity of data from other age groups. We need to further investigate the accuracy and consistency of our results, but we will continue to monitor research related to different lifestyles and depression.

## Data availability statement

The original contributions presented in the study are included in the article/**Supplementary Material**. Further inquiries can be directed to the corresponding author.

## Ethics statement

Ethical approval was not required for the study involving humans in accordance with the local legislation and institutional requirements. Written informed consent to participate in this study was not required from the participants or the participants’ legal guardians/next of kin in accordance with the national legislation and the institutional requirements.

## Author contributions

SG: Data curation, Formal analysis, Visualization, Writing – original draft. WZ: Writing – review & editing. LY: Writing – original draft. LJ: Visualization, Writing – original draft. DT: Investigation, Writing – review & editing. TZ: Writing – review & editing. BQZ: Writing – review & editing. BZ: Methodology, Project administration, Supervision, Writing – review & editing.

## References

[1] Malhi GS Mann JJ. Depression. Lancet (London England). (2018) 392:2299–312. doi: 10.1016/s0140-6736(18)31948-2 30396512

[2] Collaborators GDaIIaP. Global, regional, and national incidence, prevalence, and years lived with disability for 354 diseases and injuries for 195 countries and territories, 1990-2017: a systematic analysis for the Global Burden of Disease Study 2017. Lancet (London England). (2018) 392:1789–858. doi: 10.1016/s0140-6736(18)32279-7 PMC6227754 30496104

[3] Moussavi S Chatterji S Verdes E Tandon A Patel V Ustun B. Depression, chronic diseases, and decrements in health: results from the World Health Surveys. Lancet (London England). (2007) 370:851–8. doi: 10.1016/s0140-6736(07)61415-9 17826170

[4] Ustün TB Ayuso-Mateos JL Chatterji S Mathers C Murray CJ. Global burden of depressive disorders in the year 2000. Br J Psychiatry. (2004) 184:386–92. doi: 10.1192/bjp.184.5.386 15123501

[5] Belmaker RH Agam G. Major depressive disorder. N Engl J Med. (2008) 358:55–68. doi: 10.1056/NEJMra073096 18172175

[6] Heim C Binder EB. Current research trends in early life stress and depression: review of human studies on sensitive periods, gene-environment interactions, and epigenetics. Exp Neurol. (2012) 233:102–11. doi: 10.1016/j.expneurol.2011.10.032 22101006

[7] Hammarström A Lehti A Danielsson U Bengs C Johansson EE. Gender-related explanatory models of depression: a critical evaluation of medical articles. Public Health. (2009) 123:689–93. doi: 10.1016/j.puhe.2009.09.010 19853877

[8] Mata J Thompson RJ Jaeggi SM Buschkuehl M Jonides J Gotlib IH. Walk on the bright side: physical activity and affect in major depressive disorder. J Abnorm Psychol. (2012) 121:297–308. doi: 10.1037/a0023533 21553939 PMC3982878

[9] Boden JM Fergusson DM. Alcohol and depression. Addiction. (2011) 106:906–14. doi: 10.1111/j.1360-0443.2010.03351.x 21382111

[10] Lipton RI. The effect of moderate alcohol use on the relationship between stress and depression. Am J Public Health. (1994) 84:1913–7. doi: 10.2105/ajph.84.12.1913 PMC1615404 7998629

[11] Boden JM Fergusson DM Horwood LJ. Cigarette smoking and depression: tests of causal linkages using a longitudinal birth cohort. Br J Psychiatry. (2010) 196:440–6. doi: 10.1192/bjp.bp.109.065912 20513853

[12] Lassale C Batty GD Baghdadli A Jacka F Sánchez-Villegas A Kivimäki M. Healthy dietary indices and risk of depressive outcomes: a systematic review and meta-analysis of observational studies. Mol Psychiatry. (2019) 24:965–86. doi: 10.1038/s41380-018-0237-8 PMC6755986 30254236

[13] Frank P Jokela M Batty GD Lassale C Steptoe A Kivimäki M. Overweight, obesity, and individual symptoms of depression: A multicohort study with replication in UK Biobank. Brain Behav Immun. (2022) 105:192–200. doi: 10.1016/j.bbi.2022.07.009 35853559 PMC10499756

[14] Franzen PL Buysse DJ. Sleep disturbances and depression: risk relationships for subsequent depression and therapeutic implications. Dialogues Clin Neurosci. (2008) 10:473–81. doi: 10.31887/DCNS.2008.10.4/plfranzen PMC3108260 19170404

[15] Pandi-Perumal SR Monti JM Burman D Karthikeyan R BaHammam AS Spence DW. Clarifying the role of sleep in depression: A narrative review. Psychiatry Res. (2020) 291:113239. doi: 10.1016/j.psychres.2020.113239 32593854

[16] Thomée S Härenstam A Hagberg M. Mobile phone use and stress, sleep disturbances, and symptoms of depression among young adults–a prospective cohort study. BMC Public Health. (2011) 11:66. doi: 10.1186/1471-2458-11-66 21281471 PMC3042390

[17] Smith GD Ebrahim S. 'Mendelian randomization': can genetic epidemiology contribute to understanding environmental determinants of disease? Int J Epidemiol. (2003) 32:1–22. doi: 10.1093/ije/dyg070 12689998

[18] Burgess S Foley CN Zuber V. Inferring causal relationships between risk factors and outcomes from genome-wide association study data. Annu Rev Genomics Hum Genet. (2018) 19:303–27. doi: 10.1146/annurev-genom-083117-021731 PMC6481551 29709202

[19] Firth J Solmi M Wootton RE Vancampfort D Schuch FB Hoare E. A meta-review of "lifestyle psychiatry": the role of exercise, smoking, diet and sleep in the prevention and treatment of mental disorders. World Psychiatry. (2020) 19:360–80. doi: 10.1002/wps.20773 PMC7491615 32931092

[20] Burgess S Davey Smith G Davies NM Dudbridge F Gill D Glymour MM. Guidelines for performing Mendelian randomization investigations: update for summer 2023. Wellcome Open Res. (2019) 4:186. doi: 10.12688/wellcomeopenres.15555.3 32760811 PMC7384151

[21] Sanderson E. Multivariable mendelian randomization and mediation. Cold Spring Harb Perspect Med. (2021) 11. doi: 10.1101/cshperspect.a038984 PMC7849347 32341063

[22] Hemani G Zheng J Elsworth B Wade KH Haberland V Baird D. The MR-Base platform supports systematic causal inference across the human phenome. Elife. (2018) 7. doi: 10.7554/eLife.34408 PMC5976434 29846171

[23] Zhou W Liu G Hung RJ Haycock PC Aldrich MC Andrew AS. Causal relationships between body mass index, smoking and lung cancer: Univariable and multivariable Mendelian randomization. Int J Cancer. (2021) 148:1077–86. doi: 10.1002/ijc.33292 PMC7845289 32914876

[24] Bowden J Davey Smith G Burgess S. Mendelian randomization with invalid instruments: effect estimation and bias detection through Egger regression. Int J Epidemiol. (2015) 44:512–25. doi: 10.1093/ije/dyv080 PMC4469799 26050253

[25] Bowden J Davey Smith G Haycock PC Burgess S. Consistent estimation in mendelian randomization with some invalid instruments using a weighted median estimator. Genet Epidemiol. (2016) 40:304–14. doi: 10.1002/gepi.21965 PMC4849733 27061298

[26] Burgess S Scott RA Timpson NJ Davey Smith G Thompson SG. Using published data in Mendelian randomization: a blueprint for efficient identification of causal risk factors. Eur J Epidemiol. (2015) 30:543–52. doi: 10.1007/s10654-015-0011-z PMC4516908 25773750

[27] Hartwig FP Davey Smith G Bowden J. Robust inference in summary data Mendelian randomization *via* the zero modal pleiotropy assumption. Int J Epidemiol. (2017) 46:1985–98. doi: 10.1093/ije/dyx102 PMC5837715 29040600

[28] Yuan S Larsson SC. Inverse association between serum 25-hydroxyvitamin D and nonalcoholic fatty liver disease. Clin Gastroenterol Hepatol. (2023) 21:398–405.e4. doi: 10.1016/j.cgh.2022.01.021 35101633

[29] Liu Q He H Yang J Feng X Zhao F Lyu J. Changes in the global burden of depression from 1990 to 2017: Findings from the Global Burden of Disease study. J Psychiatr Res. (2020) 126:134–40. doi: 10.1016/j.jpsychires.2019.08.002 31439359

[30] Byrne EM Yang J Wray NR. Inference in psychiatry *via* 2-sample mendelian randomization-from association to causal pathway? JAMA Psychiatry. (2017) 74:1191–2. doi: 10.1001/jamapsychiatry.2017.3162 29094155

[31] Anda RF Williamson DF Escobedo LG Mast EE Giovino GA Remington PL. Depression and the dynamics of smoking. A national perspective. Jama. (1990) 264:1541–5. doi: 10.1001/jama.264.12.1541 2395193

[32] Gilman SE Abraham HD. A longitudinal study of the order of onset of alcohol dependence and major depression. Drug Alcohol Depend. (2001) 63:277–86. doi: 10.1016/s0376-8716(00)00216-7 11418232

[33] Błaszczyk B Straburzyński M Więckiewicz M Budrewicz S Niemiec P Staszkiewicz M. Relationship between alcohol and primary headaches: a systematic review and meta-analysis. J Headache Pain. (2023) 24:116. doi: 10.1186/s10194-023-01653-7 37612595 PMC10463699

[34] de Boer N Vermeulen J Lin B van Os J Ten Have M de Graaf R. Longitudinal associations between alcohol use, smoking, genetic risk scoring and symptoms of depression in the general population: a prospective 6-year cohort study. Psychol Med. (2023) 53:1409–17. doi: 10.1017/s0033291721002968 PMC10009403 35023464

[35] Gea A Beunza JJ Estruch R Sánchez-Villegas A Salas-Salvadó J Buil-Cosiales P. Alcohol intake, wine consumption and the development of depression: the PREDIMED study. BMC Med. (2013) 11:192. doi: 10.1186/1741-7015-11-192 23988010 PMC3765610

[36] Gea A Martinez-Gonzalez MA Toledo E Sanchez-Villegas A Bes-Rastrollo M Nuñez-Cordoba JM. A longitudinal assessment of alcohol intake and incident depression: the SUN project. BMC Public Health. (2012) 12:954. doi: 10.1186/1471-2458-12-954 23134690 PMC3526561

[37] Agabio R Trogu E Pani PP. Antidepressants for the treatment of people with co-occurring depression and alcohol dependence. Cochrane Database Syst Rev. (2018) 4:Cd008581. doi: 10.1002/14651858.CD008581.pub2 29688573 PMC6494437

[38] Zhou H Polimanti R Yang BZ Wang Q Han S Sherva R. Genetic risk variants associated with comorbid alcohol dependence and major depression. JAMA Psychiatry. (2017) 74:1234–41. doi: 10.1001/jamapsychiatry.2017.3275 PMC6331050 29071344

[39] Breslau N Novak SP Kessler RC. Daily smoking and the subsequent onset of psychiatric disorders. Psychol Med. (2004) 34:323–33. doi: 10.1017/s0033291703008869 14982138

[40] Wootton RE Richmond RC Stuijfzand BG Lawn RB Sallis HM Taylor GMJ. Evidence for causal effects of lifetime smoking on risk for depression and schizophrenia: a Mendelian randomisation study. Psychol Med. (2020) 50:2435–43. doi: 10.1017/s0033291719002678 PMC7610182 31689377

[41] Galan D Perry BI Warrier V Davidson CC Stupart O Easton D. Applying Mendelian randomization to appraise causality in relationships between smoking, depression and inflammation. Sci Rep. (2022) 12:15041. doi: 10.1038/s41598-022-19214-4 36057695 PMC9440889

[42] Bayes J Schloss J Sibbritt D. Effects of polyphenols in a mediterranean diet on symptoms of depression: A systematic literature review. Adv Nutr. (2020) 11:602–15. doi: 10.1093/advances/nmz117 PMC7231605 31687743

[43] Sala-Climent M López de Coca T Guerrero MD Muñoz FJ López-Ruíz MA Moreno L. The effect of an anti-inflammatory diet on chronic pain: a pilot study. Front Nutr. (2023) 10:1205526. doi: 10.3389/fnut.2023.1205526 37521415 PMC10381948

[44] Serra-Majem L Trichopoulou A Ngo de la Cruz J Cervera P García Alvarez A La Vecchia C. Does the definition of the Mediterranean diet need to be updated? Public Health Nutr. (2004) 7:927–9. doi: 10.1079/phn2004564 15482619

[45] Brookie KL Best GI Conner TS. Intake of raw fruits and vegetables is associated with better mental health than intake of processed fruits and vegetables. Front Psychol. (2018) 9:487. doi: 10.3389/fpsyg.2018.00487 29692750 PMC5902672

[46] Nicoli MC Anese M Parpinel M. Influence of processing on the antioxidant properties of fruit and vegetables. Trends Food Sci Technology. (1999) 10:94–100. doi: 10.1016/S0924-2244(99)00023-0

[47] Zhang D Hamauzu Y. Phenolics, ascorbic acid, carotenoids and antioxidant activity of broccoli and their changes during conventional and microwave cooking. Food Chem. (2004) 88:503–9. doi: 10.1016/j.foodchem.2004.01.065

[48] Chen TT Chen CY Fang CP Cheng YC Lin YF. Causal influence of dietary habits on the risk of major depressive disorder: A diet-wide Mendelian randomization analysis. J Affect Disord. (2022) 319:482–9. doi: 10.1016/j.jad.2022.09.109 36162666

[49] Oliván-Blázquez B Aguilar-Latorre A Motrico E Gómez-Gómez I Zabaleta-Del-Olmo E Couso-Viana S. The relationship between adherence to the mediterranean diet, intake of specific foods and depression in an adult population (45-75 years) in primary health care. A Cross-Sectional Descriptive Study. Nutrients. (2021) 13. doi: 10.3390/nu13082724 PMC8399773 34444884

[50] Malmir H Mirzababaei A Moradi S Rezaei S Mirzaei K Dadfarma A. Metabolically healthy status and BMI in relation to depression: A systematic review of observational studies. Diabetes Metab Syndr. (2019) 13:1099–103. doi: 10.1016/j.dsx.2019.01.027 31336451

[51] Silva DA Coutinho E Ferriani LO Viana MC. Depression subtypes and obesity in adults: A systematic review and meta-analysis. Obes Rev. (2020) 21:e12966. doi: 10.1111/obr.12966 31724325

[52] Moazzami K Lima BB Sullivan S Shah A Bremner JD Vaccarino V. Independent and joint association of obesity and metabolic syndrome with depression and inflammation. Health Psychol. (2019) 38:586–95. doi: 10.1037/hea0000764 PMC6626601 31120270

[53] Chu K Cadar D Iob E Frank P. Excess body weight and specific types of depressive symptoms: Is there a mediating role of systemic low-grade inflammation? Brain Behav Immun. (2023) 108:233–44. doi: 10.1016/j.bbi.2022.11.016 PMC10567582 36462595

[54] Karageorgiou V Casanova F O'Loughlin J Green H McKinley TJ Bowden J. Body mass index and inflammation in depression and treatment-resistant depression: a Mendelian randomisation study. BMC Med. (2023) 21:355. doi: 10.1186/s12916-023-03001-7 37710313 PMC10502981

[55] Gao T Xiang YT Zhang H Zhang Z Mei S. Neuroticism and quality of life: Multiple mediating effects of smartphone addiction and depression. Psychiatry Res. (2017) 258:457–61. doi: 10.1016/j.psychres.2017.08.074 28917440

[56] Wong SM Chen EY Wong CS Suen YN Chan DL Tsang SH. Impact of smartphone overuse on 1-year severe depressive symptoms and momentary negative affect: Longitudinal and experience sampling findings from a representative epidemiological youth sample in Hong Kong. Psychiatry Res. (2022) 318:114939. doi: 10.1016/j.psychres.2022.114939 36343577

[57] Pereira FS Bevilacqua GG Coimbra DR Andrade A. Impact of problematic smartphone use on mental health of adolescent students: Association with mood, symptoms of depression, and physical activity. Cyberpsychol Behav Soc Netw. (2020) 23:619–26. doi: 10.1089/cyber.2019.0257 32580574

[58] Xie H Tao S Zhang Y Tao F Wu X. Impact of problematic mobile phone use and insufficient physical activity on depression symptoms: a college-based follow-up study. BMC Public Health. (2019) 19:1640. doi: 10.1186/s12889-019-7873-z 31805915 PMC6896767

[59] Yang J Fu X Liao X Li Y. Association of problematic smartphone use with poor sleep quality, depression, and anxiety: A systematic review and meta-analysis. Psychiatry Res. (2020) 284:112686. doi: 10.1016/j.psychres.2019.112686 31757638

[60] Wei X An F Liu C Li K Wu L Ren L. Escaping negative moods and concentration problems play bridge roles in the symptom network of problematic smartphone use and depression. Front Public Health. (2022) 10:981136. doi: 10.3389/fpubh.2022.981136 36733277 PMC9886682

[61] Ohayon MM Roth T. Place of chronic insomnia in the course of depressive and anxiety disorders. J Psychiatr Res. (2003) 37:9–15. doi: 10.1016/s0022-3956(02)00052-3 12482465

[62] Zhang MM Ma Y Du LT Wang K Li Z Zhu W. Sleep disorders and non-sleep circadian disorders predict depression: A systematic review and meta-analysis of longitudinal studies. Neurosci Biobehav Rev. (2022) 134:104532. doi: 10.1016/j.neubiorev.2022.104532 35041878

[63] Baglioni C Riemann D. Is chronic insomnia a precursor to major depression? Epidemiological and biological findings. Curr Psychiatry Rep. (2012) 14:511–8. doi: 10.1007/s11920-012-0308-5 22865155

[64] Riemann D Krone LB Wulff K Nissen C. Sleep, insomnia, and depression. Neuropsychopharmacology. (2020) 45:74–89. doi: 10.1038/s41386-019-0411-y 31071719 PMC6879516

[65] Christensen H Batterham PJ Gosling JA Ritterband LM Griffiths KM Thorndike FP. Effectiveness of an online insomnia program (SHUTi) for prevention of depressive episodes (the GoodNight Study): a randomised controlled trial. Lancet Psychiatry. (2016) 3:333–41. doi: 10.1016/s2215-0366(15)00536-2 26827250

[66] Lane JM Jones SE Dashti HS Wood AR Aragam KG van Hees VT. Biological and clinical insights from genetics of insomnia symptoms. Nat Genet. (2019) 51:387–93. doi: 10.1038/s41588-019-0361-7 PMC6415688 30804566

[67] Wirz-Justice A. Diurnal variation of depressive symptoms. Dialogues Clin Neurosci. (2008) 10:337–43. doi: 10.31887/DCNS.2008.10.3/awjustice PMC3181887 18979947

[68] Murray G. Diurnal mood variation in depression: a signal of disturbed circadian function? J Affect Disord. (2007) 102:47–53. doi: 10.1016/j.jad.2006.12.001 17239958

[69] Moffoot AP O'Carroll RE Bennie J Carroll S Dick H Ebmeier KP. Diurnal variation of mood and neuropsychological function in major depression with melancholia. J Affect Disord. (1994) 32:257–69. doi: 10.1016/0165-0327(94)90090-6 7897090

[70] Germain A Nofzinger EA Meltzer CC Wood A Kupfer DJ Moore RY. Diurnal variation in regional brain glucose metabolism in depression. Biol Psychiatry. (2007) 62:438–45. doi: 10.1016/j.biopsych.2006.09.043 PMC3195370 17217926

[71] Bowen RC Mahmood J Milani A Baetz M. Treatment for depression and change in mood instability. J Affect Disord. (2011) 128:171–4. doi: 10.1016/j.jad.2010.06.040 20674035

[72] Werner-Seidler A Li SH Spanos S Johnston L O'Dea B Torok M. The effects of a sleep-focused smartphone application on insomnia and depressive symptoms: a randomised controlled trial and mediation analysis. J Child Psychol Psychiatry. (2023) 64:1324–35. doi: 10.1111/jcpp.13795 PMC10952387 36991537

[73] Alesci S Martinez PE Kelkar S Ilias I Ronsaville DS Listwak SJ. Major depression is associated with significant diurnal elevations in plasma interleukin-6 levels, a shift of its circadian rhythm, and loss of physiological complexity in its secretion: clinical implications. J Clin Endocrinol Metab. (2005) 90:2522–30. doi: 10.1210/jc.2004-1667 15705924

[74] Haug HJ. Prediction of sleep deprivation outcome by diurnal variation of mood. Biol Psychiatry. (1992) 31:271–8. doi: 10.1016/0006-3223(92)90050-A 1547300

