# Supplementary figures and images for: The potential causal relationship between various lifestyles and depression: a univariable and multivariable Mendelian randomization study
Source: Front Psychiatry. 2024 Feb 29;15:1343132. doi: 10.3389/fpsyt.2024.1343132 (PMC10937522; doi:10.3389/fpsyt.2024.1343132)

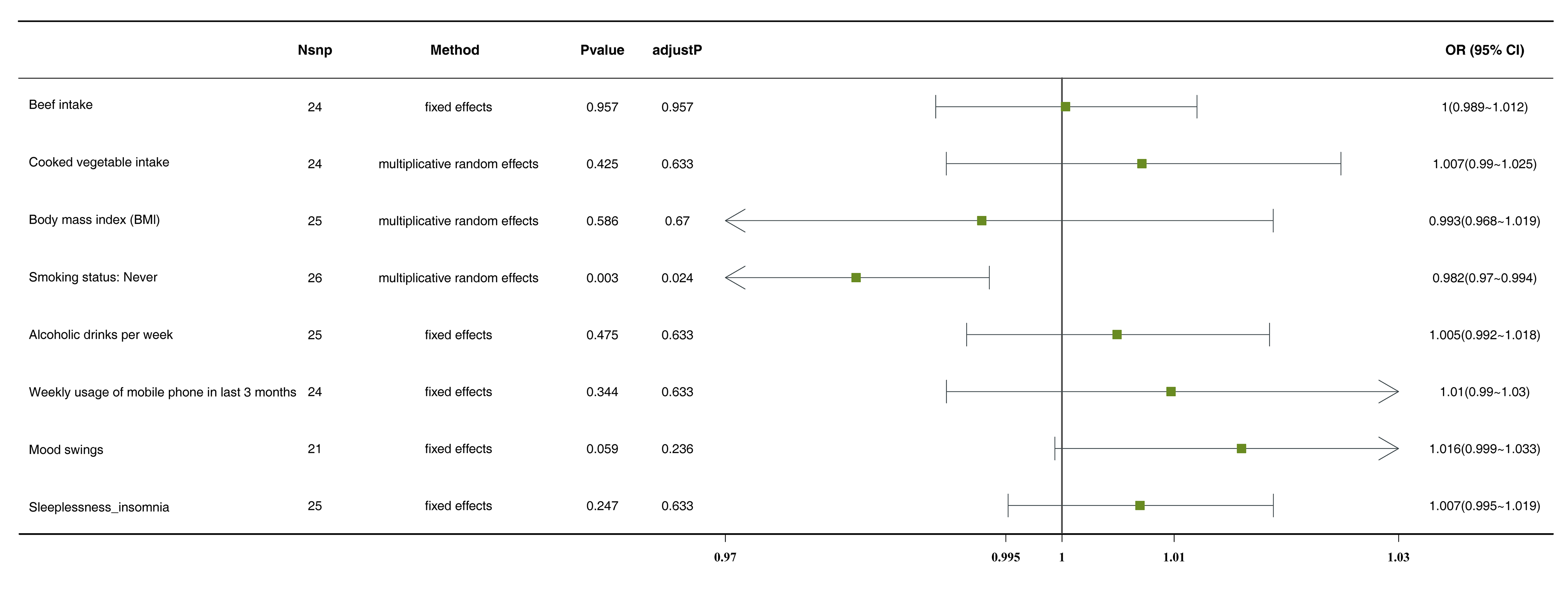

Supplement: Supplementary file 1 [file Image_1.tif]
