# Supplementary material for: The potential causal relationship between various lifestyles and depression: a univariable and multivariable Mendelian randomization study
Source: Front Psychiatry. 2024 Feb 29;15:1343132. doi: 10.3389/fpsyt.2024.1343132 (PMC10937522; doi:10.3389/fpsyt.2024.1343132)
Supplement: Supplementary file 3 [file DataSheet_1.docx]

Supplementary Material

**Supplementary** **Table 1:** The Single nucleotide polymorphisms (SNPs) list of ebi-a-GCST006944_Mood swings.

**Supplementary Table 2:** The SNPs list of ieu-b-73_Alcoholic drinks per week.

**Supplementary Table 3:** The SNPs list of ukb-a-13_Sleeplessness_insomnia.

**Supplementary Table 4:** The SNPs list of ukb-a-248_Body mass index (BMI).

**Supplementary Table 5:** The SNPs list of ukb-b-2862_Beef intake.

**Supplementary Table 6:** The SNPs list of ukb-b-8089_Cooked vegetable intake.

**Supplementary Table 7:** The SNPs list of ukb-b-17999_Weekly usage of mobile phone in last 3 months.

**Supplementary Table 8:** The SNPs list of ukb-d-20116_0_Smoking status_ Never.

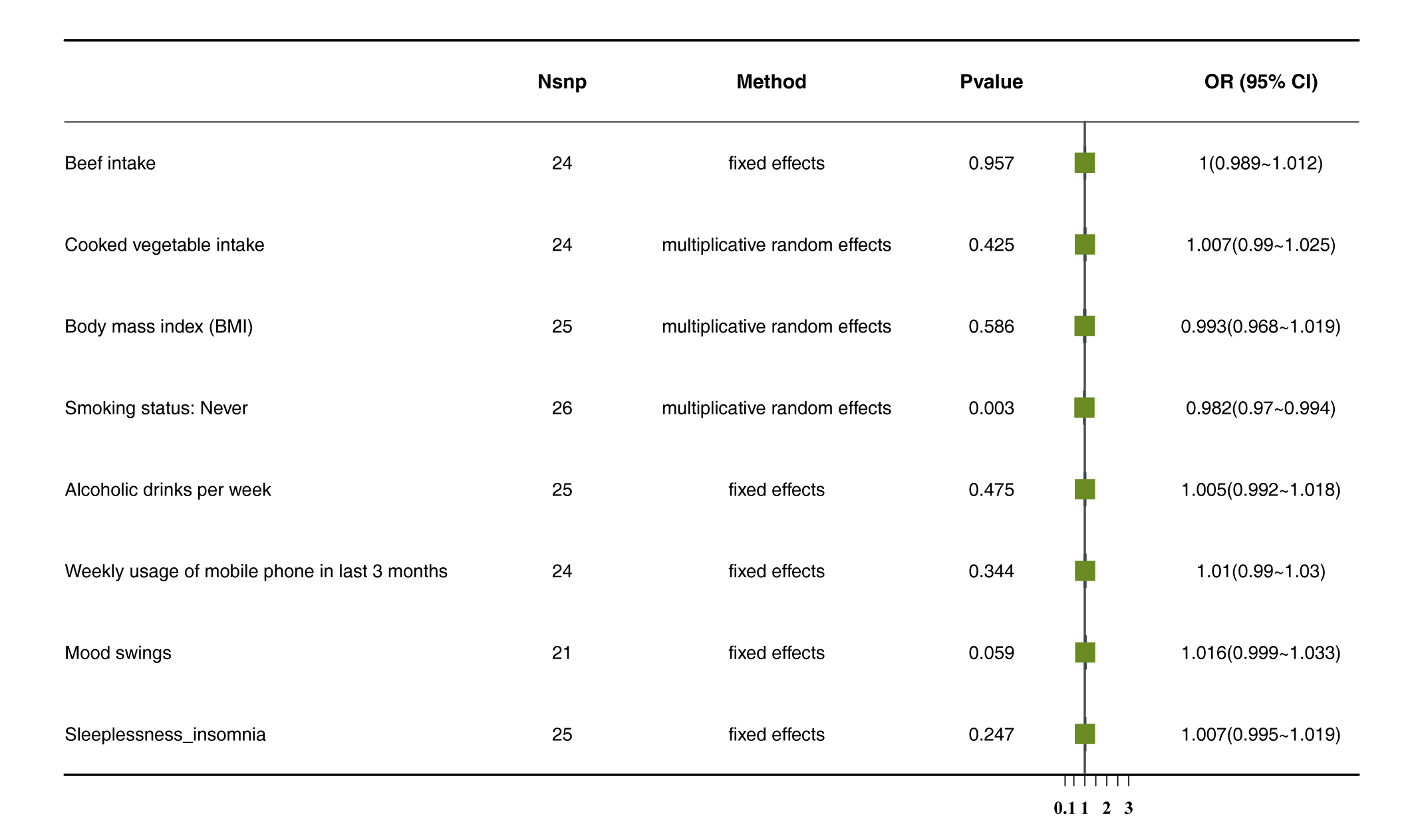


**Supplementary Figure 1.** The results of reverse MR analysis between eight lifestyles and depression.
